# A 3D-Printed Anatomical Pancreas Model for Robotic-Assisted Minimally Invasive Surgery

**DOI:** 10.3390/jfb16060207

**Published:** 2025-06-03

**Authors:** Calin Vaida, Andra Ciocan, Andrei Caprariu, Corina Radu, Nadim Al Hajjar, Doina Pisla

**Affiliations:** 1 CESTER, Technical University of Cluj-Napoca, 400114 Cluj-Napoca, Romania; calin.vaida@mep.utcluj.ro (C.V.); andra.ciocan10@gmail.com (A.C.); corinaradu@gmail.com (C.R.); na_hajjar@yahoo.com (N.A.H.); 2 Department of Surgery, “Iuliu Hatieganu” University of Medicine and Pharmacy, 400347 Cluj-Napoca, Romania; 3 Department of Internal Medicine, “Iuliu Hatieganu” University of Medicine and Pharmacy, 400347 Cluj-Napoca, Romania; 4 Technical Sciences Academy of Romania, B-dul Dacia, 26, 030167 Bucharest, Romania

**Keywords:** pancreatic phantom model, preoperative surgical planning, additive manufacturing, robotic-assisted surgical training

## Abstract

The paper presents the design, manufacturing, and evaluation of a 3D-printed pancreas phantom model used for preoperative surgical planning and surgical training. Several manufacturing and design alternatives have been explored, leading to the final solution, which consisted of a transparent 3D printed elastic shell of the pancreas, resulting in an empty volume that was filled with a custom hydrogel to ensure an anatomically realistic behaviour. Additionally, specific vascular structures were printed using elastic material and specific colours. The hollow shell of the pancreas and the vascular structures were manufactured using photopolymerisation technology. The hydrogel, which replicates the internal structure of the pancreas, was made from a custom proportion of gelatine, agar, and glycerol. The phantom model of the pancreas was assessed by the surgical team and tested using the PARA-SILSROB parallel robot designed for single-incision surgical procedures.

## 1. Introduction

### 1.1. Background and Motivation

The pancreas is a vital organ located in the superior region of the abdomen, functioning as an exocrine and endocrine gland. As an exocrine gland, the role of the pancreas is to produce digestive enzymes that are used to break down the nutrients inside the small bowel. As an endocrine gland, it secretes the hormones involved in glycemia control, such as insulin and glucagon [1].

Pancreatic cancer is considered one of the leading causes of cancer-related deaths worldwide. Although pancreatic cancer ranks as the 12th most common type of cancer worldwide, with more than 500,000 estimated cases in 2022, it is the sixth leading cause of cancer-related deaths, accounting for almost 470,000 deaths in 2022, and is still characterised by a poor prognosis [2].

**Figure 1:**
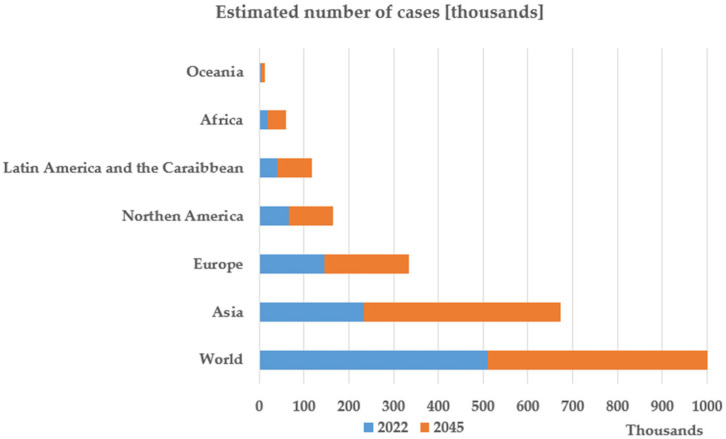
Graphical data created based on the Estimated Number of New Pancreatic Cancer Cases from 2022 to 2045 [3]. Available online: https://gco.iarc.fr/en (accessed on 5 May 2025).

Figure 1 [3] presents the estimation of the International Agency for Research on Cancer regarding the number of deaths caused by pancreatic cancer in 2045. The presented prognostic is pessimistic, showing that in 2045 the mortality rate in pancreatic cancer is expected to increase by 81.8% compared to 2022.

The disease is characterised by insidious, subclinical onset, rapid progression, and limited treatment options. Surgical resection remains the most effective treatment, offering the best overall and disease-free survival. However, in advanced stages, systemic chemotherapy and radiation become necessary [2].

Recent studies and randomised control trials, summarised in [4,5], have shown that robotic-assisted pancreatic surgery proved to be non-inferior to open or laparoscopic approach in terms of oncological outcomes to which the benefits of minimally invasive surgery are added (e.g., shorter hospital stays, less postoperative complications etc.). This motivated the team to evaluate the phantom model of the pancreas on an innovative parallel robot [6,7,8], PARA-SILSROB, designed for single-incision laparoscopic surgery, which has been already validated, as an experimental model, for oesophageal surgery [8]. The integration of both elements (the phantom model and the robot) in an advanced training tool can represent an important step forward for the education of young surgeons in complex procedures and the advancement of robotic surgery.

The aggressiveness of pancreatic cancer and its poor prognosis motivate researchers to actively explore the field in search of innovation. Due to the complexity of the surgical procedure, preoperative surgical planning and surgical training should decrease complications and improve the post-operative survival rates and quality of life. As a consequence, the motivation of this study is: (1)—the development of a phantom model of the pancreas starting from CT/MRI segmentation, which can provide an important tool for surgeons in the preplanning phase of the difficult case, and in the other hand, (2)—the resulting phantom models can be used as valuable training materials for young surgeons.

### 1.2. Importance of Preoperative Surgical Planning and Surgical Training

Accurate preoperative surgical planning is an essential step for achieving the most favourable outcomes in pancreatic surgery. Given the associated risks of pancreatic surgery, preoperative surgical planning plays a key role in minimising intraoperative risks and postoperative complications [9].

Effective preoperative planning helps to reduce the risk of intraoperative haemorrhage, superior mesenteric pedicle injury, and post-surgical complications such as pancreatic or biliary fistulas [9]. By identifying patient-specific anatomical variants, parenchyma structure, Wirsung duct and common bile duct dilation present, surgeons can establish the optimal surgical plan and minimise intraoperative decision-making under pressure, leading to shorter operative times and lower complication rates. Additionally, preoperative planning promotes better communication within surgical teams, ensuring that the members understand the complexity of the procedure and are aware of the potential challenges. A well-defined preoperative plan can also increase the confidence of the members of the surgical team [10].

Pancreatic surgical procedures are among the most technically demanding procedures in clinical practice, often associated with high morbidity rates. As such, comprehensive and structured surgical training is essential for achieving favourable patient outcomes. A retrospective study [11] analysing over 2100 pancreatoduodenectomy cases in Florida between 2002 and 2007 revealed that hospitals with active surgical residency programs reported significantly better clinical outcomes compared to those without surgical training facilities. Moreover, institutions that combined residency training with the implementation of Enhanced Recovery After Surgery (ERAS) protocols for pancreatic cancer demonstrated shorter hospital stays, reduced in-hospital mortality, and lower overall healthcare costs [12].

It is important to mention that the study revealed that surgical residency programs had a more substantial positive impact on patient outcomes than the sheer number of procedures performed annually by individual surgeons. These findings underscore the critical role of structured surgical training in improving the safety and effectiveness of complex pancreatic procedures. Furthermore, they highlight the value of specialised training tools—such as anatomically and mechanically accurate phantom models—in enhancing the preparedness and skill of surgical teams [11].

### 1.3. Importance of 3D-Printed Phantom Models

The medical imaging techniques can only provide visual information without any haptic feedback, which would be necessary for the surgeons to understand the biomechanical properties of the pancreas [13]. Consequently, there is a growing need for physical models that could replicate the pancreas’ mechanical behaviour.

Advancements in additive manufacturing have transformed surgical planning and medical education, with high potential in complex fields such as pancreatic surgery. 3D-printed phantom models enhance the diagnostic and preoperative value of medical imaging by providing detailed, patient-specific tactile representations of anatomical structures, thereby allowing surgeons to better visualise and interact with the pancreas prior to surgery [14].

The key benefit of 3D-printed models lies in their ability to improve procedural safety and clinical outcomes. By utilising patient-specific imaging data, these models support accurate assessment of tumour location, vascular involvement, and resectability. Studies have shown that incorporating phantom models into preoperative workflows can reduce operative time, decrease intraoperative complications, and enhance patient safety. Furthermore, they provide valuable tools for surgical training, offering realistic practice environments without patient risk [14].

With emphasis on pancreatic surgery, these models can have a high impact, where precise manipulation of fragile, deep-seated structures is critical. Surgical simulations using 3D-printed phantoms have been shown to improve surgeon confidence and support better intraoperative decision-making [14].

A recent 2023 study [15] demonstrated the clinical value of patient-specific pancreatic phantoms derived from CT scans. The study highlighted significant improvements in anatomical understanding and surgical planning and emphasised their role in training and patient communication. However, those models focused on visual fidelity and did not reproduce the biomechanical properties of the pancreatic tissue. The present study aims to address this limitation by developing a mechanically accurate model capable of providing realistic tactile feedback, particularly suited for robotic-assisted simulations.

Despite these advantages, accurately replicating the pancreas’s biomechanical properties remains challenging. As a soft and highly deformable organ, the pancreas is difficult to mimic using conventional 3D printing materials and methods.

Nonetheless, the integration of 3D printing into surgical planning represents a substantial advancement in personalised medicine. As additive manufacturing technologies evolve, the ability to produce functionally and mechanically accurate pancreatic phantoms is expected to improve. This study contributes to the field by evaluating various material compositions and structural designs, aiming to enhance the biomechanical realism and practical utility of 3D-printed pancreas models.

### 1.4. Objectives of the Study

The main objective of the study is to develop a realistic and easy-to-manufactured pancreas phantom model that can be used for pancreatic cancer surgery preoperative planning and surgeons’ training. To achieve the goal, the study is structured around the following objectives:

- Segmentation of CT/MRI data to design a 3D patient-specific phantom model of the pancreas with internal and external anatomical details (vascular structures and tumours).
- Optimisation of the Pancreatic Structure by generating different hollow shell structures and evaluating their mechanical behaviour.
- Generation of different internal structures and evaluate their mechanical properties.
- Development of a 3D design methodology to ensure the reproducibility and adaptability of the model for different applications.
- Evaluation of the Phantom Model for Surgical Applications.
- Evaluation of the feasibility of the phantom model in practical surgical applications based on the surgeon’s feedback.
- Comparison of the behaviour of the phantom model with the real pancreas based on surgeons’ feedback.
- Validation of the phantom model using the PARA-SILSROB parallel robot for SILS.
- Contribution to Pancreatic Surgery Research.
- Definition of a practical framework for future research and development of 3D printed soft tissue phantom models.

Accordingly, the study aims to offer a practical solution for improving the design and production of 3D printed soft-tissue phantom models, either as a fast and efficient solution to develop a preoperative model for complex cases, ready in less than 24 h or as a technique to create organ models with specific internal structures that can be used for the training of surgeons.

The paper is structured as follows. The introduction presents the importance of 3D printed models of organs with similar geometric and tactile characteristics as the human ones, motivating the study for the development of a pancreas phantom. Section 2 presents the 3D printing technology, and the materials used to create the pancreas model. It shows the iterative design which led to a model with similar characteristics to the human pancreas. Section 3 presents the results of the iterative phantom development, focused on the model design, hydrogel mixture definition and the surgical team evaluation of the printed model. An experimental setup using a SILS parallel robot is presented to illustrate the integration of the pancreas phantom in the training station. Section 4 presents the conclusions and future work.

## 2. Materials and Methods

### 2.1. Material Selection

A J5 MediJet 3D Printer from Stratasys, Ltd. (Eden Prairie, MN, USA) [16] is used for the development of the 3D-printed phantom model of the pancreas. This printer uses the PolyJet^TM^ technology which is a multi-material jetting photopolymer technology which enables the combination of different materials into a single final model. Besides the base model material (DraftWhite^TM^), Elastico^TM^ material was used [17] to print the pancreas model together with pigments to obtain any colour for the individual parts. Elastico^TM^ is a rubber-like photopolymer, ideal for advanced design and prototyping, enabling the printing of elastic parts with complex geometries, which can withstand repeated flexion and bending. By efficient combination of the base material with Elastico^TM^, the model hardness can be configured starting from Shore 50 (softest) up to Shore 95 (almost rigid) in 7 (seven) intermediate values. An initial sample (a cube with a side of 1 cm) has been printed and tested, by touch, by several surgeons to select the value that best mimics the pancreas tissue, whereas, as a general consent, the softest configuration, Elastico^TM^ Shore 50, was selected as the best option. As the stiffness of the 3D printed cube was still too high, the phantom model had to be created as an external shell with a softer internal structure (twin support walls, or a low-density filler) to reach the stiffness of the human pancreas.

### 2.2. Pancreas Phantom Model—Design and Manufacturing Considerations

For developing a realistic pancreas phantom model, a multi-step approach based on 3D designing, model prototyping and iterative design refinements was implemented. This section presents the iterative design process that was used to achieve a mechanical and anatomically accurate pancreas phantom model.

#### 2.2.1. The 3D Modelling Process of the Pancreatic Surface

The first step in designing the pancreas phantom model was to generate a realistic 3D model of the pancreas’s external surface (shell). For this purpose, an STL model of the pancreas was obtained from anonymised medical imaging data through segmentation [18] and further processed in Siemens NX 24.12 [19] where irregularities were reduced, leading to a smooth surface. At this stage, key factors taken into account included the following:

- The preservation of the anatomical shape of the pancreas.
- The assurance of a uniform and printable surface.
- The reduction of excessive surface complexity to facilitate the 3D printing process.

The segmentation process was done by the medical team using an automated segmentation method implemented in 3DSlicer, using the TotalSegmentator 2.5.0 extension. The segmentation was run on a computer with 12th Gen Intel i9-12900K, 128 GB RAM (5600 MHz) and Nvidia RTX A6000 as GPU Card.

#### 2.2.2. Creating a Reduced Test Model

To ensure a faster and smoother prototyping process while avoiding excessive material consumption, a reduced-scale test model was developed. The test model was used to test the printability of the model and to evaluate the mechanical behaviour of different structures.

**Figure 2:**
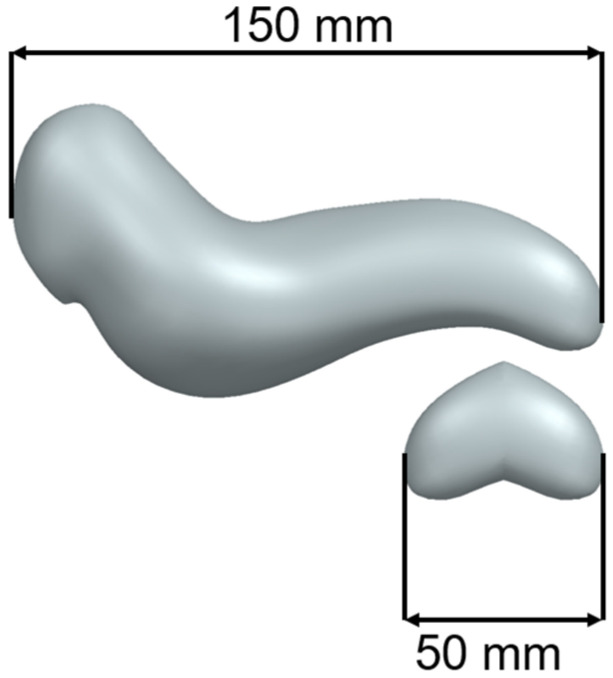
Full-Scale Pancreas Model vs. Test Model.

The test model was achieved by trimming the tail of the pancreas and mirroring it to obtain a closed volume. The main advantage of the reduced-scale test model is that prototypes can be printed quickly while consuming a reduced quantity of material. Figure 2 presents the test model for the initial printing extracted from a full-scale pancreas model developed by the authors.

#### 2.2.3. Initial Design: Vascularised Model with 1.5 mm Thick Hollow Shell with Support Material Inside

**Figure 3:**
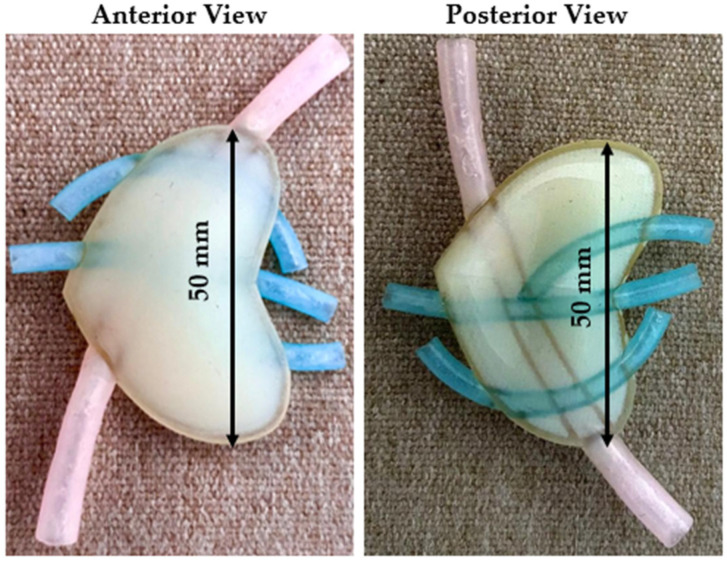
Initial model—anterior and posterior view.

The first test model consisted of a 1.5 mm thick hollow shell with the softest possible support material in the interior. Also, based on the surgeon’s suggestion, a vascular structure was added to the test model. Since there is no possibility of using a softer material than the Shore 50 one, in the first attempt, support material was used to replicate the internal structure of the pancreas. The main advantage of the support material was that it was much softer than Elastico^TM^ Shore 50. Figure 3 presents the anterior (left) and the posterior (right) view of the initial model. The purpose of the initial design was to evaluate the accuracy of the hollow shell and to have first insights regarding the model.

Conclusions: While the initial proposed model is the simplest and fastest production option, as it requires no post-processing and the entire structure is printed as an assembly, the surgeons concluded that the model did not reach the necessary softness level, which led to a new design.

#### 2.2.4. Second Design: Vascularised Model with 1.5 mm Thick Hollow Shell with Hydrogel Inside

The purpose of the second design was to test whether a hydrogel structure could replicate the mechanical behaviour of the pancreas’ internal structure.

Since the support material cannot be removed from the interior of a closed volume, especially from the blood vessels, a different approach was used. Instead of printing the entire assembly as a single part, the pancreas model was divided into 3 components: the upper half the lower half of the external shell, and the blood vessels. The three components were joined together using a strong-bound, flexible adhesive.

**Figure 4:**
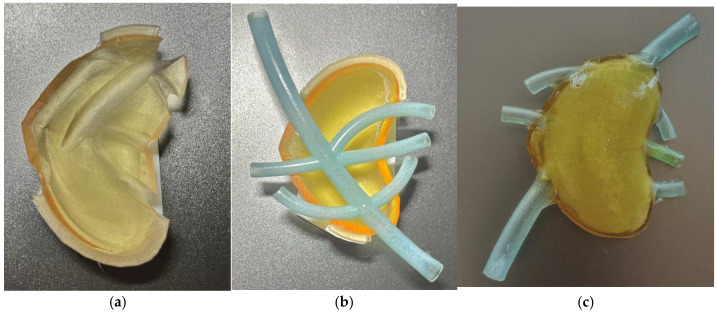
(**a**) Upper half; (**b**) lower half with vascular structure; (**c**) assembled model.

Figure 4a presents the upper half of the test model, Figure 4b presents the lower half and the vascular structure, and Figure 4c presents the assembled model, in which gelatine was used as an internal filler.

Conclusions: the second design, even though it imposes post-processing, allows the use of an internal filler for the structure which can be prepared with different configurations to lower the softness of the model to values similar to the human pancreas. Thus, the next design aimed to create the full-size model adapted for multi-part printing (to support contour bound) and the study of different hydrogel types to reach the desired softness level for the pancreas phantom.

#### 2.2.5. Final Design—Full Scale Vascularised Pancreas with 2 mm Thick Hollow Shell and Hydrogel Inside

Based on the evaluation of the second test model, which showed a mechanical behaviour that was close to that of the human pancreas, the full-scale model was designed and manufactured.

For better mechanical accuracy, the hydrogel composition was improved. It consisted of a specific combination of water, alimentary gelatine, agar, and glycogen. Each ingredient, gelatine, agar, and glycerol, plays distinct and complementary roles in achieving the mechanical and physical properties of the hydrogel. Alimentary gelatine is a protein-based hydrogel that provides a soft and elastic matrix that can mimic the viscoelastic properties of soft tissues [20]. Agar is a natural component extracted from red algae. It is used to increase the thermal stability of hydrogel [21]. Glycerol acts as a plasticiser and humectant agent, improving the flexibility of the gel while preventing dehydration [20,21,22].

To establish the correct ingredient proportions, 15 samples of material with different ratios of gelatine, water, agar, and glycerol were handed to the surgeons who decided which composition was the most accurate.

In the final design, a realistic vascular structure was added, containing the superior mesenteric artery, the superior mesenteric vein, the splenic vein, the splenic artery, and their main branches.

**Figure 5:**
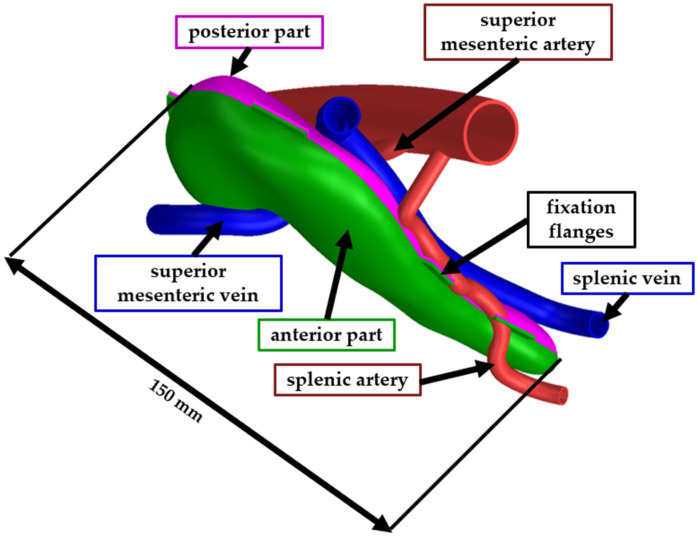
CAD model of the final phantom model.

The full-scale model was assembled similarly to the second test model. The pancreas model was trimmed in Siemens NX into two bodies (the anterior part and the posterior part) to facilitate the removal of the support material. The two bodies were assembled using a flexible adhesive after the support material was mechanically removed. The vascular structures were also printed as separate bodies and assembled using the same type of adhesive. Figure 5 presents the CAD model of the pancreas, where the green body is the anterior part, the pink body is the posterior part, the blue bodies are veins, and the red bodies are the arteries. For the ease of the assembly process, fixation flanges have been designed at the edges of the two pancreas parts. After the model was assembled, the fixation flanges were trimmed, and the surface was polished.

For the ease of the assembly process, simple flanges, 2 mm thick and approximately 4 mm wide were added to the edges of the upper and lower shell parts. The flanges were designed manually in Siemens NX to guide part placement and minimise rotational misalignment. Before assembling, both halves of the shell were frozen for 30 min to temporarily increase stiffness and prevent deformation during adhesive curing. After the two shells were assembled, the flanges were trimmed, and the surfaces were polished.

For assembling the separate printed components: the upper half, lower half, and the vascular structures, several types of adhesives have been tested. Initially, fast curing, epoxy, strong bound adhesives were tested. The primary advantage of the fast curing and instant adhesives was the ease of the joining process. However, after curing, the glue became too rigid, altering the behaviour of the hollow shell. Therefore, a flexible, strong-bound adhesive was preferred.

The adhesive used for assembling the phantom components was “Bison Poly Max Crystal Express” (Bison International B.V., Goes, The Netherlands), a transparent, flexible construction adhesive, selected based on its high bond strength and high flexibility, providing sufficient flexibility to preserve the deformability of the shell without causing stiffening at the bonded seams. It was applied in a thin, continuous layer along the edges of the shells and cured at room temperature for 24 h. A strong, fast-curing bi-component adhesive, Soudal Mitre Kit, was used for fixing the flanges together in the desired position while the flexible adhesive cured

The support material was mechanically removed from the interior of the vascular structures, which were left hollow. Future models may incorporate liquid or gel fillers to enhance anatomical realism, particularly for training simulations involving vessel dissection or exposure.

**Figure 6:**
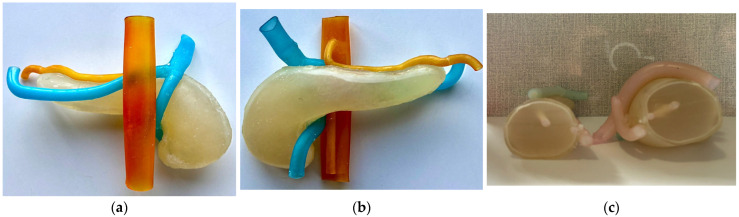
Assembled final phantom model. (**a**) Anterior view; (**b**) Posterior view; (**c**) Section cutout.

Figure 6a presents the anterior view of the assembled model with the hydrogel injected inside, Figure 6b presents the posterior view of the model, while Figure 6c presents a section cut through the phantom model.

## 3. Results and Discussion

The following sections describe in detail the development and evaluation of the 3D-printed pancreatic phantom model through an iterative design process. Section 3.1 focuses on the assessment of the initial prototype, identifying key limitations related to anatomical accuracy and mechanical behaviour. Section 3.2 introduces improvements made through the incorporation of vascular structures and hydrogel fillers to enhance tactile realism. Section 3.3 details the optimisation and validation of the final full-scale model, including surgeon feedback and material testing. Section 3.4 explores the integration of the phantom into a robotic-assisted surgical environment, demonstrating its functionality and relevance for simulation-based training and surgical planning.

### 3.1. Evaluation of the Initial Phantom Model

The evaluation of the initial pancreas phantom model was aimed at collecting the first insights regarding the mechanical and anatomical accuracy, and the suitability of the model.

The initial model was printed using a 1.5 mm thick hollow shell and internal support material. The evaluation focused on:

- **The stiffness of the structure.** The feedback from the surgeons provided valuable insights into the mechanical behaviour of the printed test model. The assessment was performed qualitatively, as the surgeons compared the tactile sensation of the model with their experience of handling the human pancreas during surgery. According to their observations, the internal structure was noticeably stiffer than that of real pancreatic tissue. As a result, redesigning the internal composition became a top priority.
- **Exterior surface smoothness.** The printed test model successfully replicated the general texture of the pancreatic capsule; however, minor surface irregularities were observed, requiring additional post-processing. The 1.5 mm thick hollow shell was evaluated qualitatively by the surgeons, who concluded that it exhibited realistic surface behaviour consistent with their surgical experience.
- **Anatomical accuracy.** Even if the structure of the pancreatic capsule was replicated, the surgeons requested more anatomical details, such as vascular structures, to be included in the model, that could further be personalised with anatomical variants in the future. The inclusion of vascular structures is essential for preoperative surgical planning and for surgical training.

To enhance the anatomical accuracy of the phantom model, alternative designs incorporating internal lattice structures were explored. The lattice-based models could replicate the overall stiffness of the pancreas. Several lattice models have been designed by varying parameters such as lattice type (tetrahedral or hexagonal), lattice mesh thickness and length. However, removing the support material from the lattice structure could not be achieved without permanent damage to the structure. Furthermore, the insertion of anatomical details such as blood vessels or tumours would not have been possible in a phantom model with a lattice structure inside.

Other alternatives would have been silicone or polyurethane-based models. Compared to those, the hydrogel-based model offers key advantages including better mechanical fidelity, better tunability, and lower production complexity. Silicone and polyurethane phantoms are too stiff compared to the pancreatic tissues. In contrast, the gelatine–agar–glycerol hydrogel used in our study, can be tuned to replicate the elastic and viscoelastic properties of the pancreas [20].

Furthermore, while silicone and polyurethane models typically require mold-based casting manufacturing, our approach allows the direct injection of the hydrogel inside the 3D-printed external shell, significantly reducing the manufacturing time and costs [20].

Building on the insights gained from the initial model evaluation, the next phase focused on developing a new printed model with enhanced mechanical behaviour, reduced stiffness, and added anatomical detail through the integration of vascular structures. These enhancements aimed to bring the model closer to the tactile and structural properties of real pancreatic tissue.

### 3.2. Evaluation of the Vascularised Model with 2 mm Thick Hollow Shell and Hydrogel Inside

The evaluation of the second test model aimed to provide insights into the hydrogel’s ability to replicate the internal stiffness of the pancreas, the accuracy of the printed vascular structures, and the feasibility of assembling the separately printed upper and lower halves, by using adhesive.

According to the feedback of the surgeons, the mechanical behaviour of the second test model was close to the mechanical behaviour of the human pancreas. The hydrogel proved to provide a similar behaviour to the target one. However, the first hydrogel composition (made only of water and gelatine) was not perfect. The structure was not stable enough on temperature variations and the elasticity was too low. Another valuable feedback from the surgeons was related to the vascular structure. The empty blood vessels significantly influenced the stiffness of the surrounding structure.

### 3.3. Evaluation of the Final Design—Full Scale Vascularised Pancreas with 2 mm Thick Hollow Shell and Hydrogel Inside

To identify the most accurate hydrogel composition, the authors designed and prepared 15 distinct samples by systematically varying the proportions of gelatine, agar, glycerol, and water. The proportions were selected based on existing literature on soft tissue-mimicking hydrogels [23] and refined through empirical testing to cover a wide spectrum of mechanical behaviours—from soft and flexible to firm and structurally stable. Each sample was then evaluated by the surgical team, who identified the formulation that most closely replicated the tactile properties of the human pancreas. Table 1 summarises the composition and expected behaviour of each tested formulation.

Based on the qualitative feedback provided by the surgeons, certain hydrogel samples were identified as either too soft or rigid, while others replicated the mechanical characteristics of pancreatic tissue at various pathological stages. Formulation 11 was consistently recognized as closely mimicking the consistency of a healthy pancreas. In contrast, Formulations 9 and 13 were found to resemble the firmer texture typically associated with pancreatitis. Additional formulations, namely 2, 5, 10, and 14 exhibited intermediate consistencies that could correspond to varying pathological conditions of the pancreas. Given that the objective of the present study was to develop a model representing a healthy pancreas, Sample 11 was selected as the final hydrogel composition.

To achieve a quantitative mechanical characterisation of the hydrogel, ten hydrogel formulations were selected from the initial 15 formulations, and their rigidity was tested using the “shear wave elastography” (SWE) method. SWE is a non-invasive ultrasound-based imaging technique that quantitatively measures tissue stiffness by computing the propagation speed of mechanically induced shear waves [24,25]. The selected samples (1, 2, 3, 5, 7, 9, 10, 11, 13, and 14) were chosen based on the surgeon’s feedback and their ability to represent a physiologically relevant range of stiffness. Each sample was tested in three replicates—samples (*n* = 3) with standardised dimensions of 100 × 100 × 20 mm to ensure statistical consistency. The hydrogel formulations that were considered relevant for the quantitative evaluation are:

- **Formulation 1**: Softest sample; baseline for very soft tissues
- **Formulation 2**: Mild elasticity—useful near the lower physiological range
- **Formulation 3**: Strong elastic behaviour—useful midpoint
- **Formulation 5**: Intermediate consistency, close to the desired texture
- **Formulation 7**: Firm, fibrotic pancreas (e.g., pancreatitis)
- **Formulation 9**: Reinforced structure but still soft
- **Formulation 10**: Strong but not brittle—can represent stiff pathology
- **Formulation 11**: Closest to the consistency of a healthy pancreas
- **Formulation 13**: Mid-firm elastic—useful for borderline fibrotic conditions
- **Formulation 14**: Durable and elastic.

**Figure 7:**
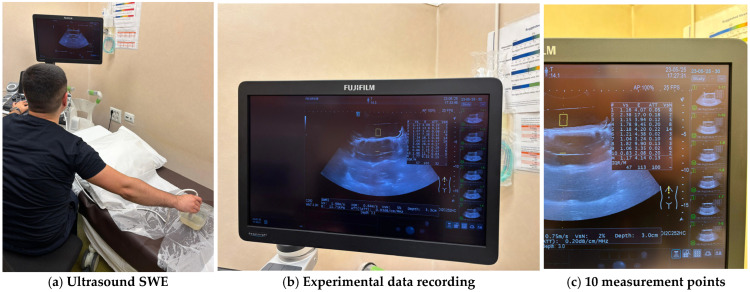
Experimental measurements of the hydrogel samples.

To achieve a continuum ultrasound environment and to eliminate boundary-related artefacts, all specimens were fully submerged in water during ultrasound testing. For each specimen, ten measurement points were selected across the surface, and SWE data were recorded using a high-frequency linear probe. All the experiments were conducted on a Hitachi (FujiFilm) Arietta 850 ultrasound machine. The system automatically computed shear wave velocity (Vs), elasticity (E), attenuation coefficient (ATT), and shear wave variability (VsN) for each measurement point. The approach allowed robust characterisation of each formulation’s elasticity profile and internal consistency while minimising operator-dependent variability and environmental noise. For each sample, the median values for the shear wave velocity Vs, the elasticity (E) and the attenuation coefficient (ATT) were automatically computed. To validate the measurement quality, the IQR/M (Interquartile Range to Median ratio) was also automatically computed as a quality index. Figure 7 illustrates the experimental determination of material properties and Table 2 presents the automatically computed values for each tested sample.

The quantitative results revealed notable differences in the mechanical behaviour of the tested hydrogel formulations. The median shear wave velocity (Vs) ranged from 0.99 m/s in Formulation 2, sample 2_3 to 2.94 m/s in Formulation 13, sample 13_1 corresponding to calculated elastic moduli (E) of 2.92 kPa and 25.95 kPa, respectively. Elasticity modulus was calculated using the formula [24]:

(1) $$E=\;3\times \rho \times {Vs}^{2}$$

where: *ρ* is the assumed density of 1000 kg/m^3^ (water density).

The median attenuation coefficient (ATT), which reflects acoustic energy loss due to absorption and scattering, varied between 0.03 dB/cm/MHz in Formulation 2, sample 2_1 and 0.36 dB/cm/MHz in Formulation10, sample 10_3, with higher values observed in samples with higher glycerol concentration.

The IQR/M metric highlights the internal dispersion of elasticity measurements across the ten measurement points per specimen. Even if most formulations exhibited acceptable IQR/M values, several specimens—particularly those with higher agar concentrations (Formulations 13 and 14)—showed elevated IQR/M ratios. The increased values may reflect localised material non-homogeneities, such as incomplete gelation, layer separation, or the presence of air bubbles. Since all the measurements were conducted in standardised conditions with fully submerged samples, consistent probe positioning, and no external compression the variations of IQR/M were not attributed to measurement artifacts. Thus, the observed IQR/M trends reflect the physical characteristics of the materials, particularly their viscoelastic response and internal structural complexity.

To validate the measurements, the values obtained for Formulation 11 (assessed qualitatively by the surgeons with similar behaviour as a healthy pancreas) were compared with data from the literature, whereas in [26], the authors reported a mean SWE value for the pancreatic head: 8.98 ± 2.46 kPa and 8.67 ± 2.67 kPa for the pancreatic body which fits perfectly with our measured elastic modulus.

To validate the anatomical and mechanical accuracy of the final pancreatic phantom model, a structured evaluation was conducted with experienced surgeons from Surgical Clinic III, “Iuliu Hațieganu” University of Medicine and Pharmacy, Cluj-Napoca. Five surgeons examined the phantom model and provided both quantitative and qualitative feedback.

The quantitative assessment focused on five key characteristics of the phantom model, rated on a scale from 1 to 10 (10 representing the highest score). The first two evaluators were senior surgeons with extensive experience in pancreatic surgery, while the remaining three were younger specialists in general abdominal surgery. The results are summarised in Table 3.

Although the evaluation cohort was limited to five participants, it consisted of experienced surgeons familiar with pancreatic surgery. The cohort size is consistent with other surgical phantom evaluation studies [14,15], where sample sizes of 3–10 participants were sufficient to obtain meaningful expert validation. The homogeneity and domain expertise of the cohort minimise variability due to experience level.

To quantify response variability, the standard deviation (SD) was calculated for each assessment question. As shown in Table 3, the scores present low to moderate variation scores (e.g., Q1: 7.8 ± 0.84; Q4: 8.2 ± 1.10), with full consistency observed in Q3 (SD = 0). The results suggest general agreement among evaluators, supporting the reliability of the descriptive analysis.

In addition to the numerical scores, qualitative feedback was collected from the surgeons, offering valuable insights into the strengths of the model and areas that could benefit from further refinement. These responses are discussed in the following section.

**Q1.**  *Based on haptic evaluation, how does the stiffness of the phantom model compare to the real human pancreas? Does it provide realistic tactile feedback when pressed/touched?*

**R1.**  *The surgeons reported that the phantom model exhibited a realistic level of stiffness comparable to that of a human pancreas. However, a slightly artificial sensation was noted, attributed to the properties of the external shell.*

**Q2.**  *How would you evaluate the anatomical accuracy of the model?*

**R2.**  *The phantom model was assessed as anatomically accurate by the surgical team, effectively replicating the macroscopic morphology of the pancreas.*

**Q3.**  *Is the model suitable for simulating minimally invasive surgical procedures either in a manual or robotic-assisted approach?*

**R3.**  *The model was considered suitable for robotic-assisted surgical simulation, particularly for planning and definition of instrument trajectories within a constrained anatomical workspace.*

**Q4.**  *What is the current utility of the phantom model in a clinical or educational setting?*

**R4.**  *The model was found to be useful for preoperative trajectory planning of surgical instruments, anatomical education, and dissection practice.*

**Q5.**  *What modifications would improve the model and bring it closer to an ideal pancreatic phantom?*

**R5.**  *To enhance the realism of the model, the surgeons recommended incorporating finer anatomical details, such as small-calibre blood vessels, the distal common bile duct, and the Wirsung duct. Additionally, they suggested the inclusion of surrounding tissues (e.g., duodenum, stomach, liver) to more accurately simulate surgical access conditions.*

The surgeon’s feedback offers valuable insights into the anatomical accuracy, clinical applicability, as well as potential improvements for future iterations of the 3D printed pancreas phantom model. This user-centred evaluation approach ensures that the model remains both clinically relevant and functionally useful. Based on the responses received, the phantom replicates the geometric and tactile characteristics of the human pancreas with sufficient accuracy to proceed to experimental validation in a robotic-assisted surgical environment.

Following the definition of the final pancreas model, a cost estimate is done to define an indicative production cost for the pancreas. Up to the point of the 3D model creation, no software costs are considered as all the processes can be done using free software tools. Table 4 illustrates the main costs to produce the pancreas model.

Based on the table above the total cost for the manufacturing of one pancreatic model is around 100 EUR and takes about two days to complete. However, these costs are lowered when more models are manufactured in parallel, with a reduction of up to 50% for ten models.

### 3.4. Experimental Evaluation of the Phantom Model

To explore the functionality of the pancreas phantom model within a robotic-assisted surgical environment, an experimental evaluation has been concluded. The experiments were performed using the PARA-SILSROB parallel robot [8].

**Figure 8:**
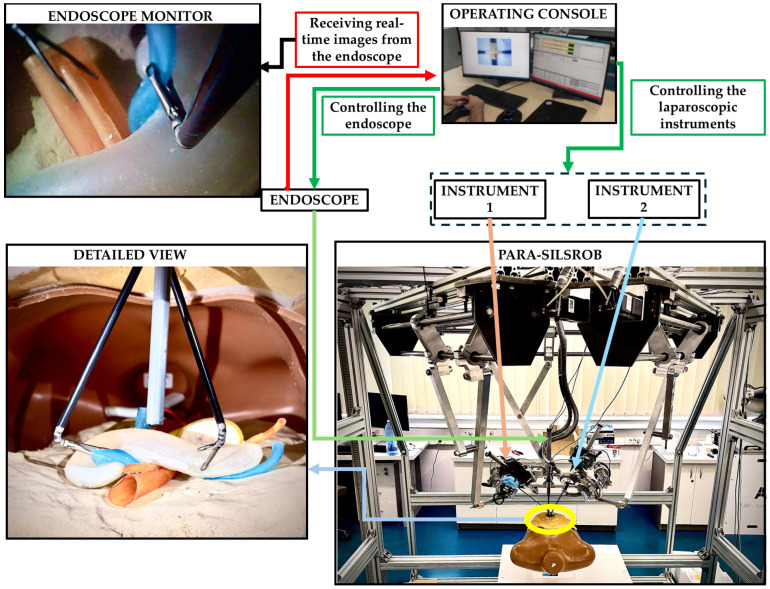
Experimental setup.

PARA-SILSROB is an innovative robotic system designed within the CESTER Research Centre, optimised for Single-Incision Laparoscopic Surgery (SILS) [8]. PARA-SILSROB has a modular structure, consisting of 6 degrees of freedom (DOF) fully parallel robot, which holds, on the mobile platform, a set of 3 independently actuated modules which hold and control the surgical instruments. The 6-DOF parallel robot consists of 3 identical kinematic chains connecting the robot’s fixed frame with the mobile platform, achieving the spatial positioning and orientation of the surgical instruments. On the mobile platform, there are three additional modules for the manipulation of surgical tools: a central one with 1-DOF for the laparoscopic camera insertion and two 3-DOF modules positioned lateral (left and right) with respect to the camera used to actuate active surgical instruments. For the procedure setup, the 6-DOF robot positions the instrument-carrying platform with respect to the SILS port, allowing the insertion of the instruments inside the patient. Once this is achieved, the platform performs solely orientation motions around the SILS port which acts as the Remote Centre of Motion (RCM). During the surgery, the camera is controlled through the reorientation of the mobile platform while the instruments are controlled independently (through their spherical modules). The control of the robot is based on a master-slave control architecture where the surgeon operates the robot (slave) from the operating console using a keyboard and two space mouses [6,7,8,9]. The PARA-SILSROB and the control console are presented in Figure 8 in an experimental environment.

Although SILS and especially robotic-assisted SILS are not common for pancreatic surgery, the experimental setup was used as a proof-of-concept scenario. The primary goal of the experiment was not to replicate a realistic surgical procedure but to evaluate the mechanical accuracy of the phantom model and its integration into a robotic-assisted surgical environment. The experimental setup is presented in Figure 8.

The pancreas phantom model was placed inside a torso trainer, in the abdominal cavity. The human torso trainer was placed on the operating table of the PARA-SILROB robotic system. A SILS port was inserted on the umbilicus level to allow access to the two clipper surgical instruments and the endoscopic camera, simulating a SILS procedure. The two surgical instruments and the endoscopic camera were manipulated by the surgeons from the operating console. The surgeons had access to the real-time images from the endoscope on the operating console’s monitors. Common pancreatic exploration and resection procedures were performed by the surgeons: retraction, palpation, and organ stabilisation. It is important to notice that no other intra-abdominal organs (e.g., intestines, stomach, liver) were placed inside the abdominal cavity of the mannequin, thus simplifying the procedure by facilitating access to the pancreatic phantom model. The experimental simplification enabled full access to the pancreatic phantom model, focusing the evaluation on the model’s mechanical properties without introducing additional anatomical interferences.

While the presented experimental setup allowed full access to the model for manipulation, it did not replicate spatial constraints imposed by overlying anatomical structures. In real surgical contexts, the presence of organs such as the duodenum, mesentery, and fat may influence tool manipulation and interaction. Future studies will incorporate these elements to simulate constrained access and evaluate the phantom’s performance under clinically realistic conditions.

The same five participants, two experienced surgeons and three younger specialists, who evaluated the phantom model previously were invited to test it within the robotic-assisted experimental setup. They performed basic laparoscopic manipulation tasks, focusing on tissue handling, instrument dynamics, and the integration of the phantom within the robotic-assisted SILS environment. In this preliminary test, two 8 mm da Vinci^R^ graspers with flexible distal heads were used [27], the focus being directed towards manipulability and tissue interaction.

To assess the interaction of the surgical team with the phantom model in the robotic-assisted experimental setup, a structured questionnaire was administered to the five participants. The questions were designed to evaluate the anatomical and mechanical fidelity of the model, the practicality of instrument handling within the SILS environment, and the model’s overall usefulness for training purposes. The questions and consolidated responses are presented below:

**Q1.**  *Does the size and external morphology of the phantom model accurately resemble that of a human pancreas?*

**R1.**  *The participants agreed that the phantom model closely replicates the typical size and external shape of a human pancreas. It was noted, however, that natural anatomical variability exists among individuals.*

**Q2.**  *Does the model reproduce realistic softness and tissue resistance during robotic-assisted manipulation?*

**R2.**  *The phantom provided a realistic tactile response during manual manipulation. While some tactile nuances are less perceptible when using robotic instruments (as the lack of haptic feedback is compensated by the visual feedback), the overall mechanical behaviour of the model was consistent with the expected properties of pancreatic tissue, supporting its suitability for simulation and training purposes.*

**Q3.**  *Was the working space around the pancreas sufficient for instrument manoeuvring in this experimental context?*

**R3.**  *The available space was considered adequate for instrument manipulation due to the absence of other abdominal organs. In real surgical scenarios, limited access to the pancreas remains a significant constraint.*

**Q4.**  *Can the phantom model be effectively used for surgical training purposes?*

**R4.**  *The model was considered highly valuable for both manual (open, laparoscopic) and robot-assisted surgical training. Unlike animal tissues, which rapidly lose mechanical properties post-extraction, the phantom model offers consistent, customisable, and repeatable conditions. Its integration into robotic platforms enhances its relevance for simulation-based training.*

**Q5.**  *What additional features would enhance the realism and utility of the phantom model?*

**R5.**  *The surgeons recommended incorporating more detailed internal anatomy, such as small blood vessels, the distal common bile duct, and the Wirsung duct. For improved realism in training, the inclusion of surrounding structures (e.g., duodenum, stomach, liver) was also recommended, particularly to simulate realistic access limitations.*

## 4. Conclusions and Further Work

This study provides a comprehensive exploration of the design, manufacturing, and evaluation of a 3D-printed pancreatic phantom model intended for surgical training and robotic-assisted applications. The results confirm the effectiveness of the proposed design methodology and additive manufacturing techniques in replicating the anatomical and mechanical properties of pancreatic parenchyma with high fidelity.

Surgeon feedback played a critical role in validating the clinical applicability of the model. Haptic assessments confirmed that the phantom closely mimics the tactile response of real pancreatic tissue, while recommendations for added anatomical detail—such as vascular structures—were successfully implemented in the final design. These iterative improvements contributed to the creation of a detailed and functional phantom, suitable for preoperative planning and simulation-based training.

The selected manufacturing approach ensured precise geometric reproduction of patient-derived anatomical structures, enhancing spatial visualisation and allowing surgeons to safely rehearse complex procedures. Furthermore, integration into a robotic-assisted environment demonstrated the model’s utility in supporting safe and effective instrument manoeuvrability.

Future work will focus on extending the phantom’s capabilities by incorporating patient-specific anatomical features derived from individual CT scans. Additional enhancements will include the integration of fine vascular networks, the distal common bile duct, and the Wirsung duct, further improving anatomical realism and expanding the model’s utility across clinical and educational contexts.

## Figures and Tables

**Table 1:** Hydrogel Composition.

	Water (%)	Gelatine (%)	Agar (%)	Glycerol (%)	Expected Behaviour
Formulation 1	88.60%	3.90%	1.40%	6.10%	Soft, flexible
Formulation 2	87.50%	5.30%	1.60%	5.60%	Moderate elasticity
Formulation 3	84.70%	6.40%	3.20%	5.70%	Strong, elastic
Formulation 4	83.40%	5.00%	3.00%	8.60%	Firm
Formulation 5	83.30%	6.30%	4.70%	5.60%	Hydrated, soft
Formulation 6	81.70%	6.50%	4.90%	6.90%	Tough, less flexible
Formulation 7	79.50%	7.90%	6.70%	6.00%	Soft but with structure
Formulation 8	83.00%	5.00%	1.50%	10.50%	Slightly firm
Formulation 9	78.80%	6.00%	6.30%	8.90%	Soft, slight reinforcement
Formulation 10	78.70%	7.00%	8.30%	5.90%	Strong but not too brittle
Formulation 11	84.10%	6.00%	3.20%	6.70%	Balanced, medium-soft
Formulation 12	85.90%	5.20%	4.60%	4.30%	Most rigid, good shape retention
Formulation 13	82.80%	5.90%	4.70%	6.60%	Firm, moderate elasticity
Formulation 14	81.30%	6.50%	6.40%	5.80%	Moderate elasticity & durability
Formulation 15	78.10%	7.70%	8.20%	5.90%	Most rigid and stable

**Table 2:** Quantitative evaluation of hydrogel samples.

Formulation	Sample	Median Vs		Median E		Median ATT	
No.	No.	[m/s]	IQR/M	[kPa]	IQR/M	[dB/cm/MHz]	IQR/M
1	1_1	1.28	46.00	4.91	107.00	0.13	41.00
	1_2	2.24	61.00	15.05	115.00	0.08	158.00
	1_3	1.40	30.00	5.87	62.00	0.15	20.00
	**average**	**1.64**		**8.61**		**0.12**	
2	2_1	1.24	50.00	4.60	115.00	0.03	245.00
	2_2	1.65	63.00	8.13	106.00	0.07	59.00
	2_3	0.99	37.00	2.92	80.00	0.08	85.00
	**average**	**1.29**		**5.22**		**0.06**	
3	3_1	1.16	59.00	4.03	116.00	0.08	139.00
	3_2	1.88	36.00	10.55	69.00	0.09	117.00
	3_3	1.73	31.00	9.00	58.00	0.09	114.00
	**average**	**1.59**		**7.86**		**0.08**	
5	5_1	1.66	69.00	8.29	136.00	0.15	96.00
	5_2	1.22	41.00	4.45	85.00	0.09	88.00
	5_3	1.81	43.00	9.77	87.00	0.14	89.00
	**average**	**1.56**		**7.50**		**0.12**	
7	7_1	1.32	20.00	5.24	42.00	0.13	28.00
	7_2	1.56	24.00	7.27	48.00	0.16	56.00
	7_3	1.38	11.00	5.73	22.00	0.13	33.00
	**average**	**1.42**		**6.08**		**0.14**	
9	9_1	2.33	34.00	16.20	62.00	0.24	17.00
	9_2	1.75	49.00	9.25	98.00	0.19	12.00
	9_3	1.77	71.00	9.50	166.00	0.06	165.00
	**average**	**1.95**		**11.65**		**0.16**	
10	10_1	1.60	52.00	7.64	113.00	0.16	47.00
	10_2	1.40	76.00	5.97	163.00	0.12	104.00
	10_3	1.21	73.00	4.38	184.00	0.36	14.00
	**average**	**1.40**		**5.99**		**0.21**	
11	11_1	1.47	51.00	6.55	115.00	0.20	23.00
	11_2	1.63	39.00	7.94	84.00	0.24	67.00
	11_3	1.74	50.00	9.11	104.00	0.17	28.00
	**average**	**1.61**		**7.86**		**0.20**	
13	13_1	2.94	18.00	25.95	36.00	0.27	31.00
	13_2	1.71	67.00	8.87	132.00	0.20	89.00
	13_3	1.80	29.00	9.72	62.00	0.13	74.00
	**average**	**2.15**		**14.85**		**0.20**	
14	14_1	1.17	47.00	4.14	113.00	0.13	100.00
	14_2	1.57	67.00	7.46	148.00	0.14	88.00
	14_3	1.78	57.00	9.54	102.00	0.15	32.00
	**average**	**1.51**		**7.05**		**0.14**	

**Table 3:** Quantitative evaluation of the phantom model by surgeons.

Assessment element	Surgeon 1	Surgeon 2	Surgeon 3	Surgeon 4	Surgeon 5	Mean	SD
Q1. Phantom model stiffness	8	7	7	9	8	7.8	0.84
Q2. Size and shape of model	8	9	8	9	8	8.4	0.55
Q3. Ease of instrument manipulation during simulation	8	8	8	8	8	8	0
Q4. Educational and clinical utility of the model	7	7	9	9	9	8.2	1.1
Q5. Potential for refinement to increase anatomical or functional realism	8	8	9	9	9	8.6	0.55

**Table 4:** Indicative costs for the pancreas model.

Procedure	Material Usage	Time Duration	Estimative Costs (EUR)
Shell model printing	Materials—144 gr	14 h 14 m	30 EUR (200 Eur per kg)
	Support—376 gr		60 EUR (150 EUR per kg)
Hydrogel	Mixture based on formulation 11	1 h	10 EUR
Assembly	Glueing	24 h	1 EUR
Hydrogel insertion in the pancreas mold	Hydrogel	0.5 h	-
Pancreas mold finish	-	12 h	-
TOTAL		51 h 45 m	Approx. 100 EUR

## Data Availability

The data presented in this study is available upon request from the corresponding author.
